# Performance of six SARS-CoV-2 RNA detection systems in symptomatic and asymptomatic pediatric and maternal patients

**DOI:** 10.2217/fvl-2021-0311

**Published:** 2021-12-15

**Authors:** Cameron A Brown, Megan H Amerson-Brown, Aliza Rahman, Charity R Webb, Ila R Singh, James J Dunn

**Affiliations:** ^1^Department of Pathology, Texas Children's Hospital, Houston, TX 77030, USA; ^2^Department of Pathology & Immunology, Baylor College of Medicine, Houston, TX 77030, USA

**Keywords:** respiratory infection, RNA extraction, RT-PCR, SARS coronavirus, surveillance

## Abstract

**Aim:** This study evaluated the real-world performance of six test systems for detection of SARS-CoV-2 in 138 pediatric and 110 adult maternal patients. **Materials & methods:** Nasopharyngeal swabs were tested directly using the Aptima™ SARS-CoV-2 (Aptima) and Simplexa™ COVID-19 Direct (Simplexa), and with Altona RealStar^®^ RT-PCR and CDC RT-PCR with nucleic acid extracted on the Roche^®^ MagNA Pure 96 (Altona-MP96) or bioMérieux EMAG^®^ (Altona-EMAG). **Results/Conclusion:** Overall percent-positive and percent-negative agreements among the six test systems were, respectively: Aptima: 94.8 and 100%; Altona-MP96: 96.5 and 99.3%; CDC-MP96: 100 and 99.3%; Altona-EMAG: 86.1 and 100%; CDC-EMAG: 98.2 and 100%; Simplexa: 87 and 99.2%. The six test systems showed agreement ranging from 92.7 (κ = 0.85) to 98.8% (κ = 0.98).

The COVID-19 pandemic, caused by SARS-CoV-2, has necessitated the rapid deployment of molecular diagnostic assays to identify infected individuals. The recommended test for diagnosis of SARS-CoV-2 infection is one that detects one or more specific viral RNA gene sequences from a respiratory tract specimen. In response to limited amounts of supplies required to perform SARS-CoV-2 detection tests, implementation of multiple types of analytic test systems, reagents and materials has been required in the pre-analytic process to meet the demand for test capacity [1]. A recent survey of 117 laboratories in the US showed that 73% were experiencing shortages of commercial testing kits for SARS-CoV-2 [2].

Part of the strategy to combat spread of the virus has been widespread diagnostic testing of symptomatic persons under investigation (PUIs); in other words, those presenting with signs and symptoms consistent with current SARS-CoV-2 infection. Testing has also been deployed at our institution to screen (SCRN) asymptomatic patients being admitted to the hospital within 3 days of a scheduled outpatient procedure or surgery and during imminent labor and delivery. Results of testing are used to determine: infection control isolation and cohorting practices; extent of personal protective equipment (PPE) utilization by healthcare workers (particularly for aerosol-generating procedures); that transplant candidates are negative for SARS-CoV-2 within 24 h of transplant; and for epidemiologic and public health investigations of close contacts of asymptomatic individuals found to be SARS-CoV-2 positive. The development and implementation of an effective screening protocol has become a central component of patient care in order to re-open healthcare facilities to patients and families [3,4]. Many laboratories often use several types of test systems each day, depending on reagent availability, urgency for test results and staffing conditions. For consistency of test results among the various systems utilized, it is important to demonstrate comparative performance characteristics, particularly clinical sensitivity.

The aim of this study was to evaluate the real-world performance of six nucleic acid amplification test systems for detection of SARS-CoV-2 RNA in pediatric and adult maternal PUI and SCRN patients. The following test systems were utilized: direct testing with the Aptima^®^ SARS-CoV-2 assay on the Hologic Panther^®^ System (Aptima [Hologic, CA, USA]) and the Simplexa™ COVID-19 Direct (Simplexa [DiaSorin Molecular LLC, CA, USA]) system and Altona RealStar^®^ SARS-CoV-2 RT-PCR kit (Altona, Hamburg, Germany) with nucleic acid extracted on the MagNA Pure 96 (Altona-MP96 [Roche®, IN, USA]) or bioMérieux EMAG^®^ (Altona-EMAG [bioMerieux, NC, USA]), and the CDC SARS-CoV-2 RT-PCR assay with nucleic acid extracted on the Roche MagNA Pure 96 (CDC-MP96) or bioMérieux EMAG (CDC-EMAG).

## Materials & methods

### Patients & samples

The test order process for SARS-CoV-2 RNA detection at our institution allowed for input of the indication for testing as either PUI (patient presenting with symptoms compatible with SARS-CoV-2 infection and/or close exposure to a known SARS-CoV-2-positive individual) or SCRN (asymptomatic patient being screened by testing in advance of an emergent or scheduled hospital procedure or at the time of admission).

A total of 248 unique patient nasopharyngeal (NP) swab specimens were collected between 28 April and 22 August 2020 and placed in 3.0 ml of M4RT viral transport media (Remel, KS, USA). Residual, retrospective sample selection was enriched to obtain an approximate 1:1 ratio of previously positive and negative specimens for PUI and SCRN patients in each population. These included 71 pediatric and 58 unrelated maternal SCRN patients and 67 pediatric and 52 unrelated maternal PUI patients. All study specimen testing was performed on the same aliquot after identical storage conditions. The initial test result originated from any of the systems mentioned in the manuscript. Those samples were stored at -80°C and after being retrospectively chosen were subjected to a single thaw and retested in all six systems at the same time. The institutional review board determined that the research study met the category of research not involving greater than minimal risk and granted a waiver of consent.

### Nucleic acid extraction systems

A specimen volume of 200 μl was extracted on the Altona-MP96 using the DNA and Viral NA small volume kit and on the Altona-EMAG using off-board lysis with specific program B according to the manufacturer’s instructions [5]. Purified nucleic acid was eluted in 50 μl of elution buffer and briefly stored on ice until an aliquot was added to the amplification reaction mixture for the Altona or CDC assays (described below).

### Nucleic acid amplification test systems

The EUA certified Aptima SARS-CoV-2 assay on the Hologic Panther System was performed according to the manufacturer’s instructions. Briefly, 500 μl of specimen was added to a Panther™ tube containing 710 μl of lysis buffer and placed on the instrument. A volume of 360 μl of the mixture was used in the target capture, transcription-mediated amplification and dual kinetic assay detection of two regions of the SARS-CoV-2 ORF1ab gene [6].

The RealStar SARS-CoV-2 RT-PCR kit is EUA certified and allows for detection and differentiation of lineage B-betacoronaviruses (B-βCoV) by targeting the E gene from B-βCoV and SARS-CoV-2-specific RNA by targeting the S gene [7]. Amplification and detection reactions were performed on the ABI 7500 instrument (Thermo Fisher Scientific, MA, USA) using 20 μl of master mix combined with 10 μl of sample eluate from either the Altona-MP96 or Altona-EMAG under the following thermocycling conditions: 55°C for 20 min, 95°C for 2 min and 45 cycles of 95°C for 15 s, 55°C for 45 s and 72°C for 15 s. Amplification curves with cycle threshold (Ct) values <45 for either target were considered positive.

The CDC 2019-novel coronavirus (2019-nCoV) real time RT-PCR diagnostic panel is EUA certified and designed for specific detection of SARS-CoV-2 RNA sequences in the N1 and N2 genes [8]. The assay also includes detection of the human RNase P gene for internal sample quality control. An eluate volume of 5 μl of nucleic acid from the CDC-MP96 or CDC-EMAG was added to 15 μl of TaqPath 1-step RT-qPCR mastermix (Thermo Fisher Scientific) and amplification was performed on the ABI 7500 (ThermoFisher) under the following thermocycling conditions: 25°C for 2 min, 50°C for 15 min, 95°C for 2 min and 45 cycles of 95°C for 3 s and 55°C for 30 s. Amplification curves with Ct values <45 for either target were considered positive.

Testing with the Simplexa COVID-19 direct assay (Diasorin Molecular) was performed according to the manufacturer’s instructions on the LIAISON^®^ MDX instrument (Diasorin Molecular). The real-time RT-PCR assay targets two different regions of the SARS-CoV-2 genome; ORF1ab and S gene [9]. Briefly, 50 μl of NP swab viral transport media and 50 μl of reaction mix were loaded into a single wedge of the direct amplification disc, sealed and loaded onto the LIAISON MDX. Samples were considered positive if one or more of the SARS-CoV-2 targets were detected by the instrument software (version 2.0).

### Statistics

In absence of a ‘gold standard’ method for detection of SARS-CoV-2, for the purposes of determining positive percent agreements (PPA) and negative percent agreements (NPA) a specimen was considered a true positive if the results of two or more of the six test systems were positive. Ct value comparisons between assays and patient populations were compared by using one SARS-CoV-2 gene target from each assay, if detected. These were Altona E gene, CDC N1 gene and Simplexa ORF1ab gene. Comparisons of PUI and SCRN patientswere performed using the Mann–Whitney test. Wilcoxon matched-pairs signed rank test was used to assess Ct values obtained from the two extraction platforms. Statistical analysis was performed using GraphPad Prism 9 (GraphPad, CA, USA).

## Results

NP swabs from 138 pediatric (67 PUI and 71 SCRN) and 110 maternal (52 PUI and 58 SCRN) patients were tested using the six different systems. At the time of initial testing, pediatric patients were 7.7 years of age on average and 47.1% were female. The average age of maternal patients was 29.2 years. For both PUI and SCRN patients, the overall PPA and NPA among the six test systems were, respectively, as follows: Aptima, 94.8% (109/115) and 100% (133/133); Altona-MP96, 96.5% (111/115) and 99.3% (133/134); CDC-MP96, 100% (115/115) and 99.3% (133/134); Altona-EMAG, 86.1% (99/115) and 100% (133/133); CDC-EMAG, 98.2% (113/115) and 100% (133/133); Simplexa, 87% (100/115) and 99.2% (130/131) (Table 1). In head-to-head comparisons, the six test systems showed overall agreement ranging from 92.7% (κ = 0.85) (Simplexa vs CDC-MP96) to 98.8% (κ = 0.98) (CDC-EMAG vs CDC-MP96) (Table 2).

**Table 1.** Overall and population-specific performance of six SARS-CoV-2 RNA detection test systems.

Population		Pos (n)	Aptima		Altona-MP96		Altona-EMAG		CDC-MP96		CDC-EMAG		Simplexa	
			PPA (95% CI)	NPA (95% CI)	PPA (95% CI)	NPA (95% CI)	PPA (95% CI)	NPA (95% CI)	PPA (95% CI)	NPA (95% CI)	PPA (95% CI)	NPA (95% CI)	PPA (95% CI)	NPA (95% CI)
PUI	Ped	33	33/33 100% (89.5, 100)	34/34 100% (89.9, 100)	33/33 100% (89.5, 100)	34/34 100% (89.9, 100)	30/33 90.9% (76.4, 96.9)	34/34 100% (89.9, 100)	33/33 100% (89.5, 100)	34/34 100% (89.9, 100)	33/33 100% (89.5, 100)	34/34 100% (89.9, 100)	31/33 93.9% (80.4, 98.2)	34/34 100% (89.9, 100)
	Mat	25	24/25 96% (80.5, 99.3)	27/27 100% (87.5, 100)	25/25 100% (87.5, 100)	27/27 100% (87.5, 100)	21/25 84% (65.4, 93.6)	27/27 100% (87.5, 100)	25/25 100% (87.5, 100)	27/27 100% (87.5, 100)	25/25 100% (87.5, 100)	27/27 100% (87.5, 100)	22/25 88% (70.0, 95.8)	26/27 96.3% (81.7, 99.3)
SCRN	Ped	32	28/32 87.5% (71.9, 95.0)	39/39 100% (91.0, 100)	30/32 93.8% (79.9, 98.2)	39/39 100% (91.0, 100)	27/32 84.4% (68.2, 93.1)	39/39 100% (91.0, 100)	32/32 100% (89.3, 100)	39/39 100% (91.0, 100)	30/32 93.8% (79.9, 98.2)	39/39 100% (91.0, 100)	27/32 84.4% (68.2, 93.1)	39/39 100% (91.0, 100)
	Mat	25	24/25 96% (80.5, 100%)	32/32 100% (89.3, 100%)	23/25 92% (75.0, 97.8)	31/32 96.9% (84.3, 99.4)	21/25 84% (65.3, 95.6)	32/32 100% (89.3, 100)	25/25 100% (86.7, 100)	31/32 96.9% (84.3, 99.4)	25/25 100% (86.7, 100)	32/32 100% (89.3, 100)	20/25 80% (60.9, 91.1)	30/30^†^ 100% (88.7, 100)
Overall		115	109/115 94.8% (89.1, 97.6)	132/132 100% (97.2, 100)	111/115 96.5% (91.4, 98.6)	131/132 99.2% (95.8, 99.9)	99/115 86.1% (78.6, 91.2)	132/132 100% (97.2, 100)	115/115 100% (96.8, 100)	131/132 99.2% (95.8, 99.9)	113/115 98.2% (93.9, 99.5)	132/132 100% (97.2, 100)	100/115 87% (79.6, 91.9)	129/130^†^ 99.2% (95.8, 99.9)

† Two (2) specimens tested invalid and were not included in the calculation.

Mat: Maternal; NPA: Negative percent agreement; Ped: Pediatric; PPA: Positive percent agreement; PUI: Symptomatic persons under investigation; SCRN: Asymptomatic screened individuals.

**Table 2.** Agreement (%) among six SARS-CoV-2 RNA detection systems.

	Aptima	Altona-MP96	Altona-EMAG	CDC-MP96	CDC-EMAG	Simplexa
Aptima	–	97.2 (κ = 0.94)	95.2 (κ = 0.90)	97.2 (κ = 0.94)	98.4 (κ = 0.97)	95.6 (κ = 0.91)
Altona-MP96	97.2 (κ = 0.94)	–	94.0 (κ = 0.88)	97.8 (κ = 0.95)	97.2 (κ = 0.94)	93.5 (κ = 0.87)
Altona-EMAG	95.2 (κ = 0.90)	94.0 (κ = 0.88)	–	93.1 (κ = 0.86)	94.4 (κ = 0.89)	94.8 (κ = 0.89)
CDC-MP96	97.2 (κ = 0.94)	97.8 (κ = 0.95)	93.1 (κ = 0.86)	–	98.8 (κ = 0.98)	92.7 (κ = 0.85)
CDC-EMAG	98.4 (κ = 0.97)	97.2 (κ = 0.94)	94.4 (κ = 0.89)	98.8 (κ = 0.98)	–	94.0 (κ = 0.88)
Simplexa	95.6 (κ = 0.91)	93.5 (κ = 0.87)	94.8 (κ = 0.89)	92.7 (κ = 0.85)	94.0 (κ = 0.88)	–

κ: Cohen’s unweighted kappa.

**Figure 1.**
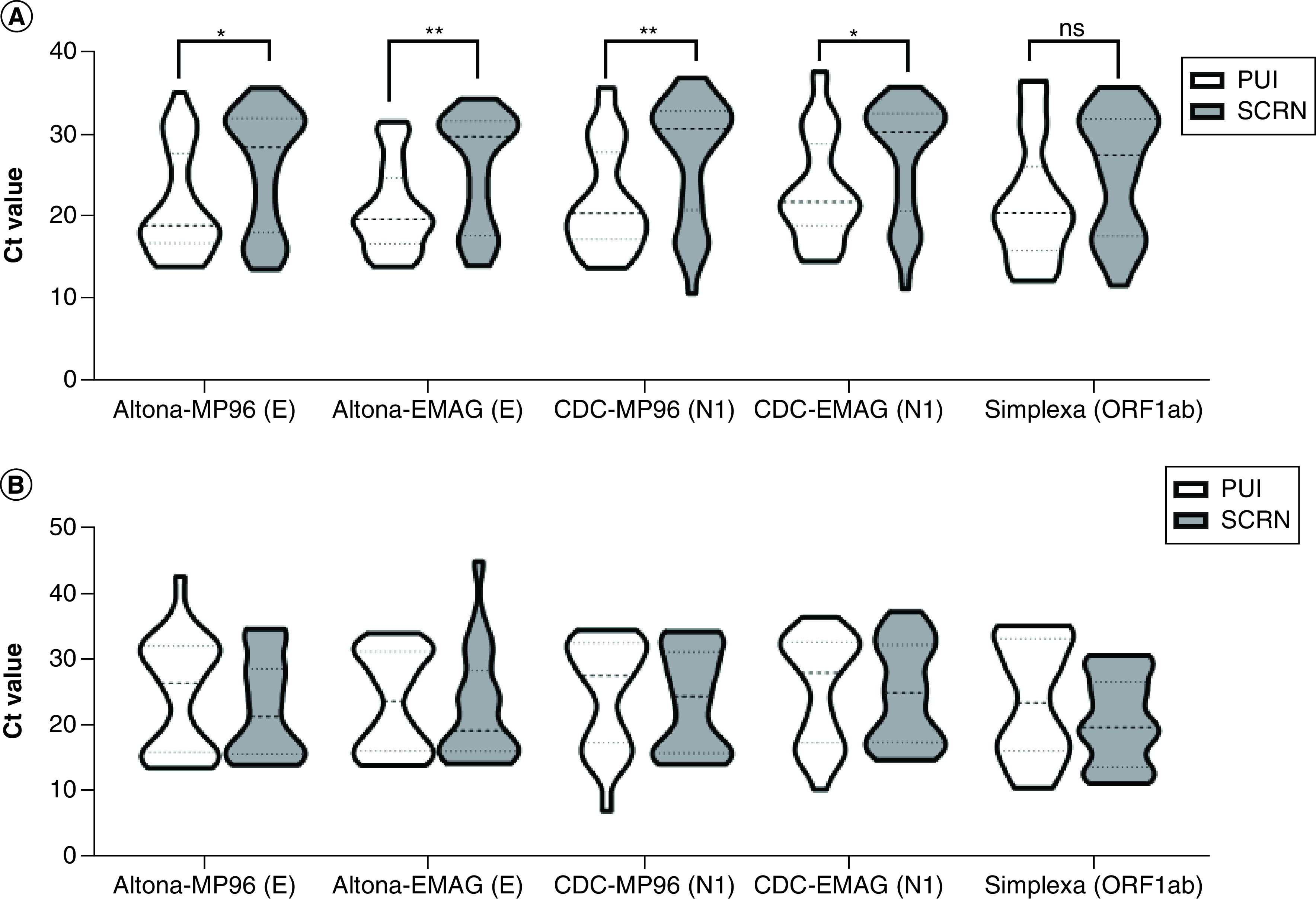
Person under investigation versus asymptomatic screened individuals patients. Real time RT-PCR (Ct) value comparison for five systems used to test for SARS-CoV-2 in **(A)** pediatric and **(B)** maternal PUI and SCRN patients using the Mann–Whitney test. *p < 0.05; **p < 0.01. Ct: Cycle threshold; PUI: Person under investigation; SCRN: Asymptomatic screened individuals.

Among pediatric PUI patients, four of the six tests detected SARS-CoV-2 in all 33 positive specimens; whereas, the Altona-EMAG and Simplexa were unable to detect viral RNA in 3 and 2 specimens, respectively (Table 1). Average Ct values from the other systems for the three Altona-EMAG false negative specimens ranged from 35.9 to 38.0, 33.5 to 36.4 and 32.2 to 35.8 for the three specimens, respectively. Simplexa false negatives in this cohort had average Ct values ranging from 35.9 to 38.0 and 30.4 to 31.2 from the other systems for the two respective specimens. In asymptomatic pediatric patients being screened for SARS-CoV-2 infection, only the CDC-MP96 test system detected virus in all 32 samples. In this group SARS-CoV-2 was not detected in four patients by Aptima testing (Ct values ranged from 37.2 to 39.0, 33.5 to 35.3, 33.5 to 33.7, 31.8 to 38.2 in the other systems), 2 by Altona-MP96 (Ct values ranged from 33.5 to 35.3 and 31.6 to 36.8 in the other systems), 5 by Altona-EMAG (Ct values ranged from 33.0 to 37.3, 37.2 to 39.0, 33.5 to 35.3, 30.8 to 33.4, 33.5 to 33.7 in the other systems), 2 by CDC-EMAG (Ct values ranged from 37.3 to 39.0 and 33.5 to 33.7 in the other systems) and 5 by Simplexa (Ct values ranged from 33.0 to 37.3, 37.3 to 39.0, 33.5 to 35.3, 33.5 to 33.7, 31.8 to 38.2 in the other systems). The differences in real time RT-PCR cycle threshold (Ct) values between SARS-CoV-2-positive PUI and SCRN pediatric patients tested with each system were statistically different except for Simplexa (Figure 1A).

The differences in Ct values among adult maternal PUI and SCRN patients tested with each system were not statistically different (Figure 1B). Among maternal PUI patients, three of the six test systems detected SARS-CoV-2 in all 25 positive specimens (Table 1). The Aptima, Altona-EMAG and Simplexa assays were unable to detect viral RNA in 1, 4 and 3 specimens, respectively. Ct values from the other systems ranged from 32.6 to 35.3 for the single Aptima false negative. Altona-EMAG false negatives had average Ct values of 32.6–35.3, 34.3–36.4, 33.2–39.9 and 32.8–36.6 in the other systems for the four respective specimens. The three false negatives on Simplexa had average Ct values from the other systems of 32.6–35.3, 30.8–33.7 and 32.8–36.6 for the three respective specimens. Only the CDC-MP96 and CDC-EMAG test systems detected SARS-CoV-2 in all 25 samples from asymptomatic maternal patients being screened for infection (Table 1). In this group SARS-CoV-2 was not detected in one patient by Aptima testing (average Cts of 34.2–36.2 in the other systems), 2 by Altona-MP96 (average Cts of 34.2–36.2 and 33.2–35.6 in the other systems), 4 by Altona-EMAG (average Cts of 31.3–34.0, 35.0–38.1, 34.2–36.2 and 33.2–35.6 in the other systems) and 5 by Simplexa (average Cts of 33.3–39.6, 33.1–39.9, 35.0–38.1, 34.2–36.2 and 33.2–35.6 in the other systems).

**Figure 2.**
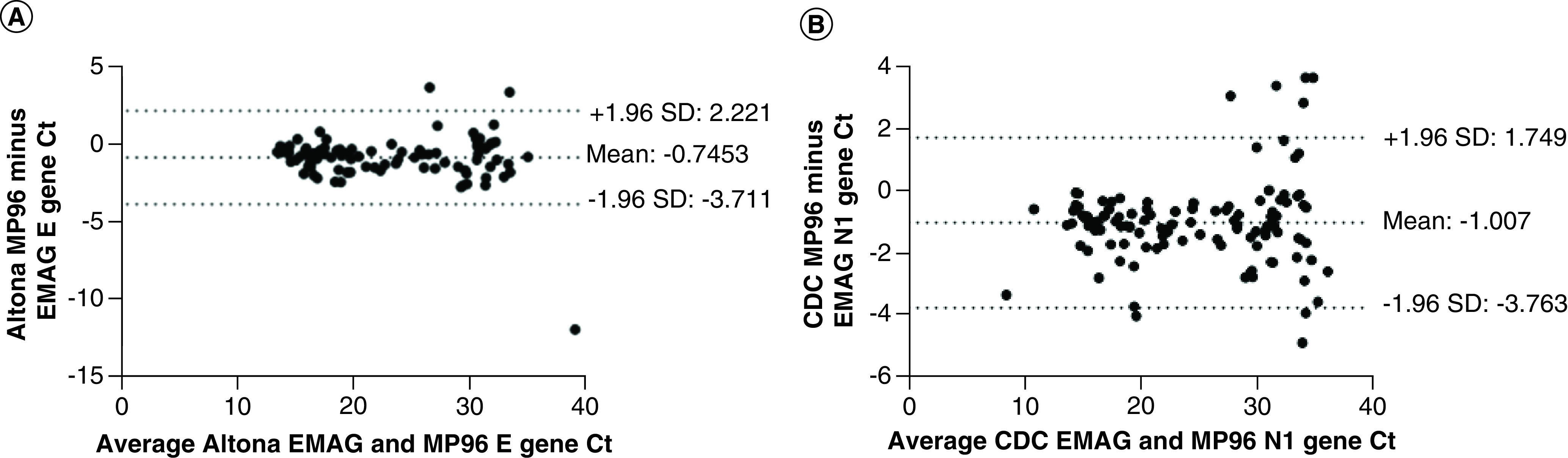
Nucleic acid extraction comparison. Bland–Altman plot for the E gene Ct values from the Altona assay extracted on **(A)** MP96 and EMAG and N1 gene Ct values from the **(B)** CDC assay extracted with MP96 and EMAG. Ct: Cycle threshold; SD: Standard deviation.

In SARS-CoV-2-positive specimens extracted on both the MP96 and EMAG, the mean bias for E gene detection using the Altona assay was -0.75 Ct (MP96 minus EMAG) with a standard deviation of 1.5 Ct (Figure 2A). The mean bias between specimens extracted on EMAG and MP96 and assessed using CDC N1 gene detection was -1.0 Ct (MP96 minus EMAG) with an standard deviation of 1.4 Ct (Figure 2B). Differences in the Ct values between extraction platforms using both assays were statistically significant (p < 0.0001, Wilcoxon matched-pairs signed rank test). Similar trends were noted for the S gene target from the Altona assay and the N2 gene target in the CDC assay (data not shown).

## Discussion

Due to supply limitations of SARS-CoV-2 tests, reagents and consumables, many laboratories have needed to employ a number of different systems and strategies to meet the demand for patient testing. Although nearly all RNA detection tests with EUA certification are intended for use with specimens obtained from symptomatic individuals, there has been an additional need to screen asymptomatic patients in order to safely manage them in healthcare settings. We assessed the performance of six nucleic acid detection test systems that have been utilized in our laboratory for both symptomatic and asymptomatic pediatric and maternal patients. The overall agreement among the tests ranged from 92.7 to 98.8% with associated kappa values of 0.85–0.98 indicating almost perfect agreement (Table 2). It was important to demonstrate that all test systems have high concordance with both PUI and SCRN patients so that we could have confidence in the final results regardless of which ones were used on a daily basis in both of these patient populations.

The six test systems tended to perform better for detection of SARS-CoV-2 with specimens from symptomatic PUIs, both pediatric (PPAs 90.9–100%) and maternal (PPAs 84–100%) patients. More false negatives were observed in asymptomatic pediatric and maternal patients being screened in advance of seeking in-person healthcare services (Table 1). Overall, false negatives in both PUI and SCRN patients were associated with Ct values at least >30 and oftentimes much higher in the other test systems able to detect SARS-CoV-2 RNA. This would suggest that the concentration of virus is low in these specimens and in some cases perhaps near or below the lower limit of detection for some assays.

Interestingly, when assessing the real time RT-PCR Ct values available from five of the test systems, there was a statistically significant difference in median Ct values for each assay for specimens from pediatric PUI and SCRN patients except for the Simplexa test (Figure 1A). In contrast, there was no difference in Ct values among maternal PUI and SCRN patients (Figure 1B). From these data it appears that symptomatic pediatric patients were more likely to have higher viral loads in the upper respiratory tract than their asymptomatic counterparts; whereas, there was no significant difference in viral load among maternal PUI and SCRN patients, a finding consistent with some previous studies [10–14]. Other studies have demonstrated a correlation between high maternal viral load and an increased rate of obstetric complications [15]. While we did assess the Ct values in this cohort and found that there was no statistical difference between symptomatic and asymptomatic pregnant women, it was beyond the scope of this paper to investigate clinical outcomes in this group.

The CDC EUA assay, Simplexa and Aptima have been assessed for analytical sensitivity by manufacturers using a panel of diluted, heat-inactivated SARS-CoV-2 provided by the US FDA (https://www.fda.gov/medical-devices/coronavirus-covid-19-and-medical-devices/sars-cov-2-reference-panel-comparative-data). The limits of detection were reported to be 18,000 NAAT detectable units (NDU)/ml, 6000 and 600 NDU/ml for the CDC EUA assay, Simplexa and Aptima, respectively. We did not assess analytical sensitivity in our study, but using clinical samples we found that the CDC assay overall detected SARS-CoV-2 in the most specimens. When nucleic acid was extracted on the MagNApure 96 and used in the CDC assay 115 of 115 samples were positive and nucleic acid from the EMAG extraction yielded 113 out of 115 positives with the CDC assay. Overall, Simplexa and Aptima detected SARS-CoV-2 in 100/115 and 109/115 specimens, respectively.

It is possible that differences in the percent positive agreements among the tests could be due to the specific SARS-CoV-2 genomic RNA sequences targeted. Assays used in this study targeted ORF1ab, E, S, N1 and N2 genes. Genomic characterization studies have determined that mutations occur more frequently in the ORF1ab and N regions with rare mutations in the S gene and none in the E gene [16]. Mutations that occur in regions commonly targeted by RT-PCR assays could influence primer/probe hybridization and result in false negatives. In addition, some studies have shown that assays that targeting the E region had a lower limit of detection [17]; whereas, detection of the RdRp region of the ORF1ab sequence in the SARS-CoV-2 genome had the highest analytical sensitivity [18,19] when compared with other targets such as the N, E and S regions.

The nucleic acid extraction process is a critical step in determining the accuracy and sensitivity of assays detecting viral RNA in clinical samples. In this study two extraction platforms, EMAG and MagNAPure 96, were used to purify nucleic acids for use in the Altona and CDC assays. Overall, the CDC-MP96 test system did detect SARS-CoV-2 in two additional specimens compared with CDC-EMAG. Similarly, the Altona-MP96 test system was able to detect SARS-CoV-2 in 12 additional samples compared with Altona-EMAG. However, among SARS-CoV-2-positive specimens there was a mean bias of -0.75 Ct for Altona E gene and -1.0 Ct for CDC N1 gene for MagNApure 96 versus EMAG, a difference which was statistically significant (Figure 2). A previous study comparing the extraction efficiency of the EMAG and MagNApure 96 for other respiratory viruses found the clinical sensitivity, specificity, positive and negative predictive values to be in the range of 90.5–100% and the average difference in Ct values obtained from samples that were extracted by the EMAG or MagNApure96 was shown to be less than one [5]. In this study, the bias between extraction methods was deemed to be clinically insignificant.

One limitation of our study was that our adult maternal population was limited to women 35 years of age and younger, so we could not assess performance of the test systems in older adults who often experience more severe manifestations of COVID-19 [20,21]. Furthermore, clinical attributes were not specifically assessed in this study, other than the test orders for PUI which by definition were deemed symptomatic patients and asymptomatic SCRN patients being tested prior to elective procedures or on admission for medical care other than for COVID-19. Clinical information on the duration of symptoms may impact differences in the observed Ct values between the groups of patients studied. The EUA instructions for use with these test systems state they should be used for individuals with signs and symptoms of COVID-19 and we applied them to a cohort of asymptomatic patients. At last, specimens were selected retrospectively to obtain nearly equivalent numbers of PUI and SCRN samples in the pediatric and maternal groups. Although they were tested on all systems after identical storage conditions, this could have biased the findings.

## Conclusion

This evaluation of six test systems for qualitative detection of SARS-CoV-2 RNA in clinical NP specimens from pediatric and maternal patients showed that there was good overall agreement but false negatives were not uncommon, particularly for asymptomatic patients being tested for screening purposes. This is an important limitation to be aware of for potential widespread use of these assays for applications such as this in the future.

**Summary points**

- Overall agreement for SARS-CoV-2 detection among the six nucleic acid amplification test systems was high (κ = 0.85–0.98).
- The overall percent positive agreement (PPA) was greater than 90% for all systems except the Diasorin Simplexa (PPA = 87%).
- The six test systems tended to perform better for detection of SARS-CoV-2 with specimens from symptomatic persons under investigations, both pediatric (PPAs: 90.9–100%) and maternal (PPAs: 84–100%) patients.
- More SARS-CoV-2 false negatives were observed in asymptomatic pediatric and maternal patients being screened in advance of seeking in-person healthcare services.
- Symptomatic pediatric patients had significantly lower SARS-CoV-2 RT-PCR cycle threshold (Ct) values compared with asymptomatic pediatric patients.
- Symptomatic and asymptomatic maternal patients did not have significantly different Ct values in the SARS-CoV-2 RT-PCR assays.
- The sensitivity of SARS-CoV-2 detection was higher overall when nucleic acid extraction from specimens was performed using the MagNApure96 compared with the EMAG system.
- Among SARS-CoV-2-positive specimens extracted using the MagNApure 96 or EMAG platforms with Ct values obtained with either the Altona (E gene) or CDC (N1 gene) assays, the mean bias was -0.75 to -1.0 Ct for MagNApure 96 versus EMAG.
